# Myelodysplastic Syndrome After Anti-CD19 Chimeric Antigen Receptor T-cell Therapy: A Case Series

**DOI:** 10.7759/cureus.44677

**Published:** 2023-09-04

**Authors:** Armaan Dhaliwal, Soumiya Ravi

**Affiliations:** 1 Department of Internal Medicine, University of Arizona College of Medicine, Tucson, USA

**Keywords:** cytokine release syndrome (crs), ccus, mds, car-t, dlbcl

## Abstract

The utility of CD19-targeted chimeric antigen receptor T-cell (CAR-T cell) therapy in the management of refractory/relapsed B-cell malignancies has increased tremendously in recent times. In addition to cytokine release syndrome (CRS), neurotoxicity, and infections, CAR-T cell patients develop cytopenias, with about 15% of the patients continuing to have severe cytopenias up to three months after treatment. Retrospective reviews have reported the development of myelodysplastic syndrome (MDS) in patients undergoing CAR-T cell therapy. Here, we describe four cases of MDS and/or clonal cytopenias of undetermined significance (CCUS), developing after CAR-T cell therapy.

A retrospective review of four patients with relapsed/refractory B-cell lymphomas treated with CD19-directed autologous CAR-T cell was conducted at our institution.

The median age was 72.5 years (range 63-76). Three of the four patients had double-hit diffuse large B-cell lymphoma (DLBCL). The median number of lines of therapy before CAR-T cell was three. Only one patient had a prior autologous stem cell transplant (ASCT). The median time to diagnosis of MDS/CCUS from CAR-T cell therapy was three months. Two cases of CCUS diagnosed were at one- and two-month post-CAR-T cell, and two cases of MDS were diagnosed at 10 and 26 months. None of the patients had dysplastic clones before the initiation of CAR-T cell therapy. Only one patient was found to have CCUS-developed CRS post-CAR-T cell requiring treatment with tocilizumab and steroids. Three patients showed complete response, with one showing a very good partial response. All the patients were in remission with no additional therapies post-CAR-T cell. One patient died secondary to COVID-19-related complications.

Four patients with prolonged cytopenias were found to have either MDS or CCUS after CAR-T cell therapy. Two CCUS cases underwent bone marrow evaluation early in the course of cytopenias and may develop into MDS, acute myeloid leukemia (AML), or myeloproliferative neoplasm over time. Our retrospective case series review, compared to previous studies, constitutes of patients with no prior clonal hematopoiesis-related cytogenetic abnormalities, fewer lines of therapy, and only one patient with previous hematopoietic stem cell transplantation (HSCT). Based on the upcoming data and our review, a bone marrow biopsy with next-generation sequencing (NGS) is imperative in patients with prolonged cytopenias after CAR-T cell therapy. A diagnosis of CCUS/MDS in these cases can help guide treatment.

## Introduction

Anti-CD19 chimeric antigen receptor T-cell (CAR-T cell) therapy has revolutionized the treatment landscape of relapsed refractory large B-cell lymphoma and B-cell acute lymphoblastic leukemia. Cytokine release syndrome (CRS) and immune effector cell-associated neurotoxicity are acute adverse effects of CAR-T cell therapy. Acute cytopenia occurs following lymphodepleting therapy of CAR-T cell therapy and in association with CRS peak with a proportion of patients continuing to have severe cytopenia after three months of infusion [1]. Late hematologic toxicity was found to be more common in patients with a prior history of stem cell transplantation and high-grade CRS, but the mechanism underlying the pathogenesis of prolonged cytopenia is unknown. The late adverse events (>90 days) after CAR-T cell infusion include hypogammaglobulinemia, infections, and secondary malignancies including myelodysplastic syndrome (MDS) [2]. Chemotherapy and/or radiotherapy leading to the development of MDS or acute myeloid leukemia (AML) is widely known. No secondary malignancies associated with retroviruses or lentiviruses that are employed in CAR-T cell production have been reported to date. There is a theoretical risk of insertional mutagenesis in using these vectors due to the phenomenon of replication-competent viruses [3,4].

There is emerging evidence that MDS can be the underlying etiology of prolonged cytopenia in patients after CAR-T cell administration. In this case series, we present a series of four patients who developed prolonged cytopenia after CAR-T cell therapy for relapsed/refractory B-cell lymphoma. Our series aims to demonstrate the presence of clonal cytopenias of undetermined significance (CCUS) and MDS in this patient population.

## Case presentation

Patient one

A 74-year-old female presented to the ED complaining of abdominal pain, vomiting, and fever. Her CT abdomen showed mesenteric lymphadenopathy, which was laparoscopically excised, and the pathology revealed grade 1-2 follicular lymphoma positive for CD20, BCL2, BCL6, CD10, and Ki-67 40% and negative for CD5, CD23, cyclin D1, and FISH rearrangement for BCL2 but not for BCL6 or cMYC. Immunohistochemistry (IHC) was negative for cMYC. She achieved a partial response with a combination of rituximab and lenalidomide but developed new cervical lymphadenopathy, which was found to be a double hit (BCL2 and MYC rearrangement) diffuse large B-cell lymphoma (DLBCL) on biopsy. She received six cycles of DA-R-EPOCH, and the repeat PET-CT revealed a right-sided cervical lymph node with a standard uptake value of 33.7.

The biopsy of the cervical lymph node demonstrated DLBCL with BCL2 translocation, no MYC translocation, and gain of MYC. Thereafter, three cycles of rituximab, ifosfamide, carboplatin, and etoposide (R-ICE) were administered, and the clinical course was complicated by neutropenic fever. Eventually, she received a lymphodepleting chemotherapy combination of fludarabine and cyclophosphamide, followed by tisagenlecleucel CAR-T cell infusion. Her post-CAR-T cell course was complicated by grade 2 CRS, which required four doses of tocilizumab. She underwent a bone marrow biopsy two months after CAR-T cell therapy which revealed no lymphoma involvement, normal cellularity with erythroid predominant trilineage hematopoiesis and <1% blasts, and mild trilineage dysplasia with 12% ring sideroblasts. Flow cytometry showed no monotypic B-cell, antigenically aberrant T-cell, or myoblast population. Karyotype and FISH study were normal. Next-generation sequencing (NGS) demonstrated the presence of TP53 and DNMT3A mutations which, combined with the presence of pancytopenia at the time, led to the diagnosis of CCUS. Four months after CAR-T cell therapy, her CT chest/abdomen/pelvis indicated complete remission. The patient is currently 15 months post-CAR-T cell therapy and is in complete remission; her one-year bone marrow biopsy showed persistent TP53 mutation with normocellular bone marrow and trilineage hematopoiesis. She continues to be neutropenic with WBC ranging from 2 K to 2.7 K and an absolute neutrophil count (ANC) from 900 to 1200.

Patient two

A 76-year-old male diagnosed with DLBCL refractory to six cycles of rituximab, cyclophosphamide, doxorubicin, vincristine, and prednisone (R-CHOP) and two cycles of R-ICE presented to the ED with a large right-sided pleural effusion and perivertebral mass. He underwent thoracentesis, and pleural fluid cytology was negative for malignancy, but flow cytometry was positive for CD10 B-cells. IHC of a perivertebral mass biopsy was positive for CD10, CD20, BCL2, and BCL6 and negative for CD3, CD6, CD30, and cyclin D1. Ki-67 was found to be 75%. There was a recurrence of the pleural effusion after a few months. Additionally, abnormal adenopathy in the right hemithorax, right and left internal mammary chains, retrocrural, and common iliac regions was observed on imaging. He was started on an off-label trial of venetoclax and polatuzumab, but the pleural effusions continued to worsen. Consequently, lymphodepletion with fludarabine and cyclophosphamide followed by tisagenlecleucel anti-CD 19 CAR-T cell infusion was administered. The patient achieved a complete response observed on PET-CT 30 and 90 days post-CAR-T cell infusion. He continued to be cytopenic and underwent a bone marrow biopsy 10 months after CAR-T cell therapy which revealed no residual lymphoma, variable bone marrow cellularity (5-50%) with markedly reduced granulopoiesis, and no morphologic evidence of dysplasia. Flow cytometry showed no monotypic B-cell, antigenically aberrant T-cell, or myoblast population. A normal karyotype and FISH profile were detected, which were normal in the pre-CAR-T cell stage as well. FISH MDS revealed 7q deletion, and NGS was positive for TP53 and SF3B1 mutations. FISH MDS and NGS testing were not done in the pre-CAR-T cell setting. The patient was diagnosed with therapy-related MDS. His counts continue to be stable with a WBC count in the range of 0.9 to 2.1 K with an ANC of 370-900. The patient is currently two years and 10 months post-CAR-T cell infusion. The patient continues to be in complete remission. His MDS is asymptomatic and is monitored closely in the clinic with labs once a month.

Patient three

A 77-year-old male presented with groin swelling and eventually developed renal failure. He was found to have mesenteric lymphadenopathy which led to left ureteric obstruction causing left hydronephrosis requiring stent placement by urology. Post-stent, his MRI revealed extensive mesenteric and retroperitoneal lymphadenopathy along with lesions in the sacrum and bilateral iliac bones. He underwent an inguinal lymph node excisional biopsy which showed grade 2 follicular lymphoma with a proliferation rate of 40-50%. IHC was positive for PAX5, CD10, BCL6, and BCL2. A month later, a PET-CT demonstrated cervical, axillary, mediastinal, mesenteric, retroperitoneal, and inguinal adenopathies and multiple FDG avid axial and appendicular skeleton osseous lesions. He received six cycles of bendamustine-rituximab combination, but the mesenteric lymph nodes continued to increase in size along with the development of new periaortic, right para-aortic, and postcaval lymph nodes. A bone marrow biopsy was performed which revealed progression to DLBCL with CD20-, PAX5-, CD30-, and BCL2-positive B-cells. FISH panel showed BCL2 rearrangement/gain of BCL6, CCDN1, MALT1, and gain but no rearrangement of cMYC. A core needle biopsy of the right inguinal lymph node signified DLBCL germinal center subtype with MYC and BCL2 rearrangement. Therapy with dose-adjusted rituximab, etoposide, prednisone, cyclophosphamide, and doxorubicin (DA-R-EPOCH) was initiated and continued for six cycles with a follow-up PET-CT revealing minimal response and development of new lymphadenopathies. Subsequently, fludarabine-cyclophosphamide lymphodepleting chemotherapy accompanied by tisagenlecleucel infusion was undertaken. PET-CT 30 days after CAR-T cell therapy showed a partial response. Bone marrow biopsy conducted at the same time revealed no morphologic or immunophenotypic evidence of residual involvement by B-cell lymphoma, a variably cellular bone marrow with trilineage hematopoiesis. Flow cytometry showed no monotypic B-cell, antigenically aberrant T-cell, or myoblast population. Karyotype and FISH study was normal. NGS demonstrated TET2 mutation. His blood counts stayed persistently low, and a diagnosis of CCUS was made. The patient continued to have prolonged cytopenias up to 10-month post-CAR-T cell infusion with WBC in the range of 1.5-2.1K with an ANC ranging from 700 to 1200. The patient, unfortunately, developed community-acquired pneumonia 10 months post CAR-T infusion with worsening respiratory failure. The patient became severely neutropenic, requiring granulocyte colony-stimulating factor. The patient chose less aggressive measures and was, therefore, managed with broad-spectrum antibiotics and high flow nasal cannula but ultimately died from hypoxic respiratory failure.

Patient four

A 63-year-old male with a history of DLBCL in remission after eight cycles of CHOP presented with liver failure a decade after his last chemotherapy. His MRI abdomen revealed hepatic lesions besides enlargement of occipital, axillary, portohepatic, peripancreatic, and retroperitoneal lymph nodes. A liver biopsy was performed which revealed DLBCL germinal center subtype + for CD5, CD10, MYC-, and Ki-67 of 90%. He was started on RCyP, but the lesions progressed significantly in a few days resulting in the switch to ICE. The chemotherapy was modified to exclude ifosfamide and etoposide to be administered at 50% of the dose due to hyperbilirubinemia. The patient achieved a partial response to the therapy after three cycles, and ultimately, a plan was made to perform an autologous stem cell transplant (ASCT) after BEAM conditioning. His disease continued to progress on the PET-CT. Eventually, he underwent lymphodepletion chemotherapy with fludarabine and cyclophosphamide followed by tisagenlecleucel infusion. He achieved a complete response confirmed on the PET-CT but continued to have pancytopenia. A bone marrow biopsy was performed 26 months after CAR-T cell therapy and did not reveal any lymphoma, but it was concerning for hypocellularity with MDS appearance. The bone marrow showed 50% cellularity with trilineage dysplasia, 11% ring sideroblasts, and no increase in blasts. Flow cytometry showed no monotypic B-cell, antigenically aberrant T-cell, or myoblast population. The karyotype was abnormal for 5q and 7q deletions. FISH was normal and FISH MDS was positive for 5q and 7q deletions. NGS revealed TP53 mutation, concerning for MDS. A few months after the biopsy, the patient contracted SARS-CoV-2-related pneumonia and died due to COVID-19-associated complications.

The summary of the different outcomes and characteristics of each of the four DLBCL cases is shown in Tables 1-2.

**Table 1:** Outcomes of each of the four DLBCL cases post CAR-T cell therapy DLBCL: diffuse large B-cell lymphoma; ASCT: autologous stem cell transplant; MDS: myelodysplastic syndrome; CCUS: clonal cytopenias of undetermined significance; CAR-T cell: chimeric antigen receptor T-cell; CRS: cytokine release syndrome; R-EPOCH: rituximab, etoposide, prednisone, cyclophosphamide, and doxorubicin; R-ICE: rituximab, ifosfamide, carboplatin, and etoposide; R-CHOP: rituximab, cyclophosphamide, doxorubicin, vincristine, and prednisone; ICE: ifosfamide, carboplatin, and etoposide; R-ECHOP: rituximab, etoposide, cyclophosphamide, doxorubicin, vincristine, and prednisone; CHOP: cyclophosphamide, doxorubicin, vincristine, and prednisone; CMR: complete metabolic response

Lymphoma type	Prior ASCT	MDS or CCUS diagnosis	Time to MDS/CCUS diagnosis	Response to CAR-T cell	CRS grade	CAR-T cell dose and construct	Lines of chemotherapy prior to CAR-T cell
DLBCL	No	CCUS	2 months	CMR	1	3.7x10^8^ (Kymrlah)	3; rituximab-ienalidomide, R-EPOCH, R-ICE
DLBCL	No	MDS	10 months	CMR	-	2.4x10^8^ (Kymrlah)	3; R-CHOP, ICE, venetoclax and polatuzumab
DLBCL	No	CCUS	1 month	Partial near CMR. Lunago score of 4	-	1.8x10^8^ (Kymrlah)	2; bendamustine and rituximab, R-ECHOP
DLBCL	Yes	MDS	26 months	CMR. Lunago score of 2	-	2.3x10^8^ (Kymrlah)	2; CHOP, ICE

**Table 2:** Genetic and tissue characteristics of each of the four DLBCL cases CAR-T cell: chimeric antigen receptor T-cell, MDS: myelodysplastic syndrome, BM: bone marrow, NGS: next-generation sequencing High-grade FISH was employed to improve the sensitivity of detecting MYC rearrangements together with BCL2 and/or BCL6 rearrangements in all four cases of DLBCL

Case	Flow cytometry	Karyotype	Double-hit MYC and BCL2 rearrangement	FISH panel	BM biopsy post-CAR-T cell	NGS post-CAR-T cell
1	Pre- and post-CAR-T cell showed no monotypic B-cells, antigenically aberrant T-cells, or myoblast populations	Pre- and post-CAR-T cell showed a normal karyotype	Yes	FISH high-grade lymphoma panel: normal pre- and post-CAR-T cell. FISH MDS panel not done at any point	Variably cellular and shows trilineage hematopoiesis with no evidence of residual/persistent lymphoma. There are no blasts and overt dysplasia present	TP53 mutation on chromosome 17 with c.589G>A VAF-5.15%. DNMT3A mutation on chromosome 2 with c.2580>A VAF-3.15%
2	Pre- and post-CAR-T cell showed no monotypic B-cells, antigenically aberrant T-cells, or myoblast populations	Pre- and post-CAR-T cell showed a normal karyotype	Yes	FISH high-grade lymphoma panel: normal pre- and post-CAR-T cell. Post-CAR-T cell FISH MDS panel deletion of 7q (seen in therapy-related MDS). FISH MDS panel not done pre-CAR-T cell	Normocellular with erythroid predominant trilineage hematopoiesis and <1% blasts. Mild trilineage dysplasia.	DNMT3A mutation on chromosome 2 with C.2098A>G and VAF of 4.95%. TP53 on chromosome 17 with c.535C>T VAF of 4.55%
3	Pre- and post-CAR-T cell showed no monotypic B-cells, antigenically aberrant T-cells, or myoblast populations	Pre- and post-CAR-T cell showed a normal karyotype	Yes	FISH high-grade lymphoma panel: normal pre- and post-CAR-T cell. FISH MDS panel not done at any point	Variably cellular marrow with trilineage hematopoiesis and mildly left-shifted myeloid maturation	TET2 mutation on chromosome 4 with c.623delC VAF of 4.22%
4	Pre- and post-CAR-T cell showed no monotypic B-cells, antigenically aberrant T-cells, or myoblast populations	Pre-CAR-T cell showed a normal karyotype, post-CAR-T cell showed deletions of 5q and 7q, consistent with MDS with a poor prognosis	No	FISH high-grade lymphoma panel: normal pre- and post-CAR-T cell. FISH MDS post-CAR-T cell showed 5q and 7q deletions, consistent with MDS. FISH MDS panel not done pre-CAR-T cell	50% cellularity with trilineage dysplasia, 11% ring sideroblasts, and no increase in blasts	TP53 mutation on chromosome 17 with c.370_371insT VAF of 23.72%

## Discussion

The patients discussed in the series have numerous risk factors for the development of MDS/CCUS like advanced age, prior chemotherapies, prior hematopoietic stem cell transplantation (HSCT), and lymphodepleting therapies before CAR-T cell. HSCT done a year prior to CAR-T cell therapy increases the risk of the development of persistent cytopenia independent of the presence of MDS/CCUS due to the inability of the marrow to recover completely after HSCT to mount an appropriate response under hematopoietic stress. MDS/AML occurring after chemotherapy or radiation exposure has been shown to have a worse prognosis than primary disease [5,6]. The risk of developing MDS after ASCT was found to be 19.8 % at 10 years [7]. Neutropenia, thrombocytopenia, and anemia are frequently observed adverse effects (94%, 80%, and 51%, respectively) after CAR-T cell infusion [8,9]. Persistent grade 3 or 4 thrombocytopenia and/or neutropenia is seen in 40-50% of the patients beyond four weeks after CAR-T cell therapy with 15% presenting with severe cytopenia after three months [1].

Shouse et al. published a case series of four patients who developed MDS after CAR-T cell therapy [9]. Strati et al. reported four cases of MDS which were diagnosed after the administration of the CAR-T cell therapy. The development of MDS was attributed to the prior chemotherapies and not to the CAR-T cell infusions in the report [10]. Unfortunately, myeloid driver somatic mutations were not tested prior to lymphodepleting chemotherapy to validate the hypothesis. Cordeiro et al. conducted a study to assess the long-term effects in 86 patients with relapsed/refractory B-cell malignancies who were treated with CAR-T cell therapy and found four cases of MDS [2]. A case of MDS development at 10 months following CAR-T cell therapy has also been reported with the patient receiving two additional lines of therapy after CAR-T cell due to relapse [11]. The similarities and differences between our patients and previous case series are summarized in Table 3.

**Table 3:** Comparison among similar studies that have studied post-CAR-T cell MDS/CCUS development CAR-T cell: chimeric antigen receptor-modified T-cell, ASCT: autologous stem cell transplant, MDS: myelodysplastic syndrome, CCUS: clonal cytopenias of undetermined significance, CRS: cytokine release syndrome, DLBCL: diffuse large B-cell lymphoma

Parameters	Shouse et al. [8]	Strati et al. [9]	Our series
Median age	74 years (range 57-76)	-	72.5 years (range 63-76)
Type of malignancy	DLBCL		DLBCL
Median lines of therapy prior to CAR-T cell	5	5	3
Patients with prior ASCT	4	1	1
Median time to MDS/CCUS diagnosis	3 months	13.5 months	6 months
Response to CAR-T cell	1 partial, 3 complete		4 complete
Pre-CAR-T cell mutations/dysplasia	1	Unknown	Unknown
CRS post-CAR-T cell	2		1
Patients who relapsed	2		None
Pre-CAR-T cell somatic mutations	1	2	None

Patients with persistent cytopenia not explained by standard tests or concomitant diseases require a bone marrow evaluation to rule out CCUS/MDS. Treatment for CCUS is not required, but a close follow-up is recommended due to the increased risk of developing into overt MDS as it represents an early stage in leukemic transformation [12].

A prospective study showed CCUS patients to have a 14 times higher probability of developing myeloid neoplasms compared to patients with no clonal disorder. Specifically, the presence of a single mutation of TET2 or DNMT3A increased the risk of progression to MDS by 50% in five years [13].

## Conclusions

The patients described in the series continue to have a complete response to CAR-T cell therapy. Long-term follow-up of patients receiving CAR-T cell is necessary to monitor for incidence of MDS/CCUS, especially in prolonged cytopenic patients. Further investigations need to be undertaken to establish a connection between CAR-T cell therapy and MDS/CCUS to allow timely management of these patients before they progress to AML. Recognizing patients with MDS/CCUS mutations before CAR-T cell can aid in the prediction of the development of these disorders post-CAR-T cell therapy.
